# Circular Design and Functionalized Upcycling of Waste Commodity Polystyrene via C-H Activation Using Microwave-Assisted Multicomponent Synthesis

**DOI:** 10.3390/polym15143108

**Published:** 2023-07-21

**Authors:** Shegufta Shetranjiwalla, Claire Cislak, Kevin M. Scotland

**Affiliations:** 1 School of Science and the Environment, Grenfell Campus, Memorial University of Newfoundland, Corner Brook, NL A2H 5G4, Canada; 2 Chemistry Department, Trent University, Peterborough, ON K9K 0G2, Canada; clairecislak@trentu.ca (C.C.); kevinscotland@trentu.ca (K.M.S.)

**Keywords:** polymer functionalization, circular design, waste commodity polystyrene, multicomponent reactions, C-H activation, sol–gel catalyst, polymer upcycling

## Abstract

The inefficient reuse and recycling of plastics—and the current surge of medical and take-out food packaging use during the pandemic—have exacerbated the environmental burden. This impels the development of alternative recycling/upcycling methods to pivot toward circularity. We report the use of the Mannich three-component coupling reaction for the modification of polystyrene (PS) recovered with a 99.1% yield from waste food containers to form functionalized nitrogen and oxygen-rich PS derivatives. A series of functionalized PS with increasing moles of formaldehyde (F) and morpholine (M) (0.5 × 10^−2^, 1.0 × 10^−2^, and 2.0 × 10^−2^ mol) was achieved using a sol–gel-derived Fe-TiO_2_ catalyst in a solvent-free, microwave-assisted synthesis. Modified polymers were characterized with viscometry, ^1^H NMR, ^13^CNMR (DEPT) FTIR, XPS, UV, and TGA. Functionalization scaled with an increasing ratio, validating the 3CR approach. Further functionalization was constrained by a competing oxidative degradation; however, the varying hydrogen bond density due to nitrogen and oxygen-rich species at higher ratios was shown to compensate for molecular weight loss. The integration of the N-cyclic quaternary ammonium cations exhibited the potential of functionalized polymers for ion-exchange membrane applications.

## 1. Introduction

Post-consumer waste plastic is difficult to recycle because the transport, separation, and reprocessing are typically more intensive and expensive compared to manufacturing from virgin resources [1]. Leakages in the various waste collection and recycling systems lead to plastics entering the environment [2]. Less than 10% of the plastics that are retained in the system are recycled globally, whereas 12% are incinerated [2], reinforcing environmental pollution and energy consumption. The surge of single-use plastics (SUPs) use during the pandemic, including masks, gloves, medical containers, and packaging, intensified this situation. Furthermore, the food service industry saw the consumption of SUP packaging increase [3], and the fees and bans on the use of SUP take-out food containers, plastic grocery bags, packaging, and utensils were either postponed, rescinded, or suspended when used in online food deliveries [4]. The reversal in consumer attitudes toward negative environmental impacts of SUP in food packaging during the pandemic has manifested in an increase in plastic waste found on shorelines [3]. This trajectory is compounded by the forecasted annual growth rate of 6.8% for the online food delivery sector by 2024 as the economy rebounds after the pandemic [5,6]. Single-use food containers produced from foamed polystyrene are among the top ten items found on international shorelines [7] and have seen less success in municipal recycling efforts [4,7]. Foamed or expanded polystyrene from food containers is inherently recyclable [8] and dissolution processes have been reported [9,10,11]. However, the main barrier to the municipal recycling of these materials is its low density and its high tendency to granulate, contaminating the other recyclable plastics during transport [8,12]. This deters convenient separation and reprocessing during recycling. Additionally, recycled polymers risk molecular weight degradation and loss in mechanical properties, effectively downcycling the polymer [13,14]. More recent efforts toward the facile depolymerization of polystyrene to monomeric styrene [15] have been reported, but depolymerization products are yet to be fully used as precursors to new polymers [16]. Therefore, a circularity-oriented framework that keeps the post-consumer expanded polystyrene in the value chain, upcycles the polymer to commercially viable products such as benzoic acid and other value-added products [17,18,19,20], and aims to reduce materials and energy use during the process [21] compared to the production from virgin resources is required [13].

Functional derivatives of polystyrene have great value and application in water purification systems as ion-exchange resins or in biomedical applications as solid-phase support for peptide synthesis (Merrifield resin) [22], bovine serum separation, and purification or catalysis [23]. They are also actively used in organic synthesis [24], functionalized nanoparticles [25], electroactivity, and the emission and absorption of light [26], as well as for realizing green chemistries, including solvent-free synthesis, [27] click chemistry [28,29], cross-coupling reactions [30], ionic liquids grafting [31], laser and ultraviolet (UV)-based methods [32], and microwave-assisted synthesis [33]. However, these functional materials are synthesized from chloromethylated polystyrene synthesized from divinylbenzene [34,35,36], and functionalization occurs on the aromatic ring [37] due to the reactivity at this site [35,38]. C-H activation has recently gained attention for imparting precise functionality and tailor properties of commodity polymers not accessible by traditional syntheses. This chemistry builds on advances in radical and photo chemistry; however, these also occur at the C(sp^2^)-H aromatic sites using virgin monomers [14,38,39]. C(sp^3^)-H activation at benzylic positions is well reported for late-stage modifications in small-molecule syntheses, but few radical [40,41] and metal-mediated [42] polystyrene modifications have been reported [37]. Although three-component (3CR) or multicomponent reactions (MCR, Passerini, and Ugi) have also been conducted successfully for post-polymerization syntheses, [43,44] the (A^3^) Mannich 3CR between an amine, aldehyde, and alkyne to synthesize propargylamine occurs via transition-metal-catalyzed C-H activation of the alkyne [45,46]. Solid-phase Mannich reactions with aldehyde, amines, and ‘active-hydrogen’ compounds also provided high yields when resin-bound alkynes were investigated [47]. Model multicomponent aromatic and benzylic Mannich reactions through C-H bond activation using Bronsted acid [48] and transition metal catalysis [49], including Cu [50], have also been established. These reactions involved the in situ generation of the imine without the exclusion of air or moisture and produced high yields [48,51]. However, this chemistry—which leverages the susceptibility and the reactivity of the benzylic C-H [52] via activation in polymeric molecules such as polystyrene for post-polymerization functionalization—has not been explored to the best of our knowledge. Since the formation of the imine is independent of the metal-catalyzed C-H bond activation, it is expected that this approach should be applicable to the multicomponent Mannich transformation in polystyrene through sp^3^ C-H activation. Microwave-assisted Mannich reactions have also shown efficient syntheses conditions, reducing the reaction time by 97% and producing competitive product yields in the formation of substituted 4-hydroxyacetophenone derivatives [53].

We report the unique three-component reaction between polystyrene recovered from food containers, morpholine (a secondary amine), and formaldehyde via C-H activation using a sol–gel-derived iron-doped titania catalyst in a solvent-free, microwave-assisted synthesis to investigate post-polymerization upcycling. The polymers were structurally characterized with nuclear magnetic resonance (NMR), Fourier transform infrared (FTIR), and ultraviolet (UV), and a molecular weight determination of the recovered and modified polystyrene polymers was performed using solution viscosity measurements. 

## 2. Experimental Section

### 2.1. Materials

Polystyrene (PS) from waste foam ‘clamshell’ restaurant take-out food containers were gathered from receptacles intended for landfills. To ensure waste polystyrene polymer was considered, only pieces branded with resin recycling identification code “6” and “PS” were collected. These were washed with soap and water and fully air-dried. Methyl ethyl ketone (MEK, Caledon Labs, Georgetown, ON, Canada), hexanes (reagent grade, VWR), toluene (reagent grade, Fischer Scientific, Hampton, NH, USA), hydrochloric acid (HCl, 37%, Aldon, Waukegan, IL, USA), morpholine (C_4_H_9_NO, 99%, Fischer Scientific), ethanol (99% reagent grade), formaldehyde (HCOH, 37% in H_2_O with 10–15% methanol stabilizer), sodium methoxide (NaOCH_3_), titanium tetraisopropoxide (Ti[(OCH(CH_3_)_2_]_4_, 98%), Pluronic P123 (PEO_20_-PPO_70_-PEO_20_), anhydrous iron trichloride (FeCl_3_), and deuterated chloroform (CDCl_3_) were all purchased from Sigma Aldrich (Oakville, ON, Canada) unless otherwise indicated and used as received. Danby 800-watt microwave (model DMW753W) was purchased from Best Buy (Peterborough, ON, Canada).

### 2.2. Polystyrene Recovery

The washed and cleaned PS containers were cut into small pieces (~1 cm × 1 cm). PS foam pieces (1 g) were dissolved in MEK solvent (5 mL) and stirred at room temperature for 30 min [54]. Following the reverse precipitation approach, hexanes as a non-solvent for precipitation was added dropwise in a 1: 3.3 (MEK: hexanes) ratio to the stirring polystyrene solution. PS was precipitated out from the solution, filtered, and dried under vacuum at 50 °C until constant weight was recorded (Figure S1 in the Supporting Information (SI)). Optimization of the dissolution methods, PS concentration and solvent: non-solvent ratios are provided in the Supporting Information (Table S1). 

### 2.3. Synthesis of Catalyst

The catalyst was adapted from a previously reported procedure [45]. Briefly, 0.09 mol of sodium methoxide in 25 mL of toluene was added to a round bottom flask fitted with a condenser and suspended in a paraffin oil bath. The solution was refluxed for 30 min before adding 0.045 mol of Pluronic P123 in an additional 25 mL of toluene to the flask. The reaction was allowed to reflux for 4 h to form the sodium salt of P123. To this mixture was added 0.09 mol of FeCl_3_, and the mixture was further refluxed for 9 h. NaCl was allowed to precipitate from the cooled solution overnight. The Fe-P123 mixture dissolved in toluene was extracted and washed twice with 20 mL toluene on reflux for 30 min to wash the sediment. The toluene was dried under vacuum at 95 °C for 3 h. The porous titania solid support was also prepared as previously reported using sol–gel synthesis [45]. HCl (0.13 mol, 37%) was added slowly dropwise to titanium (IV) isopropoxide (0.07 mol) at 0 °C. The reaction continued to stir at room temperature for 30 min. To this solution, Fe-P123 mixture (50 mg) dissolved in 1.04 mL of ethanol with 0.01 mol of 37% HCl was added. The reaction mixture was placed in a humidity chamber at 60% relative humidity for 24 h. The mixture was then dried at 60 °C for 12 h. The dried catalyst precursor was calcined at 450 °C for 30 min at a heating rate of 2 °C/min until all P123 templating agent was burned off. The Fe-doped titania (TiO_2_) catalyst was recovered as a fine brownish-black powder with some yellow and red inclusions indicating the presence of the anatase phase as the predominant phase with some rutile phase of TiO_2_ in the catalyst (Image I1 in the Supporting Information). The catalyst was characterized for Brunauer–Emmett–Teller (BET) surface area and pore measurements (Figure S2), FTIR (Figure S3a and Table S2 in the Supporting Information), and Raman spectroscopy (Figure S3b). The values and spectra agreed with those reported in literature [45,55,56,57]. 

### 2.4. Functionalization of Polystyrene

Polystyrene (PS) recovered from waste food containers was reacted with formaldehyde (F) and morpholine (M) in three PS:F:M weight-to-volume ratios. The weight of PS was kept consistent at 3.0 g (1.7 × 10^−5^ mol) for all modified polymers and was considered 1 for the variations. Volumes of formaldehyde and morpholine corresponding to 0.5 × 10^−2^ mol, 1.0 × 10^−2^ mol, and 2.0 × 10^−2^ mol were selected for modification (Table 1). In the first three-component reaction (3CR), recovered polystyrene (1.7 × 10^−5^ mol, 3.0 g) was added with formaldehyde (0.5 × 10^−2^ mol, 0.2 mL), morpholine (0.5 × 10^−3^ mol, 0.4 mL), and 0.05 g of Fe-TiO_2_ catalyst in a borosilicate Erlenmeyer flask, and was placed in the microwave at 850 watts for 5 min with incremental intervals of 5, 10, and 20 s with constant stirring after the first, second, and the final two minutes of heating, respectively. This modified PS was denoted as MPS1. Modified polymers MPS2 and MPS4 had twice and four times the F:M mole ratio with PS, respectively, compared to MPS1 (Table 1). The MPS samples were purified by soaking in 20 mL acetone in a sealed vial, stirred at 63 rpm at ambient for 22 h, and filtered to remove maximum amount of catalyst. Between 45% and 75% of the catalyst were recovered during filtration whereas small particles possibly remained embedded in the entangled polymer chains. Higher amounts of the catalyst were recovered in MPS4 than in MPS1. The recovered catalyst was not tested for further activity in this work. MPS samples were isolated as pink solids. The structure and morphology of the MPS polymers were comprehensively examined by ^1^H NMR, ^13^C NMR, FTIR, XPS, and UV spectroscopies, and the thermal properties were determined by TGA. 

### 2.5. Characterization Techniques

Fourier transform infrared (FTIR) spectroscopy was performed on a Thermo Scientific Nicolet 380 FTIR spectrometer (Thermo Electron Scientific Instruments, LLC, Waltham, MA, USA) equipped with a PIKE MIRacle™ attenuated total reflectance (ATR) system (PIKE Technologies, Madison, WI, USA). The spectrum was acquired using 64 scans at a resolution of 4 wavenumbers. All spectra were recorded at ambient temperature. The spectra were normalized against the absorbance of the band at 1601 cm^−1^ for the C-C bond vibrations of the aromatic ring. 

Nuclear magnetic resonance spectroscopy measurements (^1^H NMR (proton) and ^13^C (carbon) NMR) were recorded on a 500 MHz spectrometer (Bruker Advance III 400 spectrometer (BrukerBioSpin MRI GmbH, Karlsruhe, Germany) using a 5 mm BBO probe at 25 °C. Samples were prepared by dissolving approximately 30 mg of sample in 0.6 mL of CDCl_3_ solvent. The ^1^H NMR spectra were acquired for 4 s, collected over an 8000 Hz spectral window, with 24 transients, 64,000 data points, proton pulse of 5.35 µs, and 10 s delay. ^13^C NMR spectra were acquired for 2 s, collected over a 31,000 Hz spectral window, with 520 transients 130,000 data points, 4.5 µs pulse, and 1 s relaxation time. The ^13^C DEPT NMR spectra were acquired for 2 s, collected over a 31,000 Hz spectral window, 128 transients, 4 µs pulse, and 1 s delay.

Raman spectra were acquired using a Renishaw inVia Raman Microscope with a 532 nm laser source using 0.5% lase power. The spectra were collected with a 15 s exposure time, 10 accumulations, and a binning setting of 3. 

Ultraviolet absorption spectra were collected using Horiba Duetta^TM^ spectrofluorometer combined absorbance and fluorescence spectrometer with a built-in CCD detector. The samples were prepared by dissolving 0.02 g of sample in 4 mL of toluene and tested in a quartz cuvette. The spectrum for toluene as a solvent blank was subtracted from the final UV spectra of polystyrene and the functionalized polymers. 

Nitrogen adsorption experiments were carried out to determine the surface area and pore size distribution of the carbon materials using a Micromeretics TriStar II Plus system. Typically, 0.05–0.1 g of sample was vacuum degassed for 24 h at 110 °C prior to adsorption. Data analysis was performed with Micromeretics MicroActive software (version 5.02).

X-ray photoelectron spectroscopy (XPS) analyses were carried out on the solid samples with a Kratos AXIS Supra spectrometer using a monochromatic Al K(alpha) source (15 mA, 15 kV). The instrument work function was calibrated to give a binding energy (BE) of 83.96 eV for the Au 4f7/2 line for metallic gold and the spectrometer dispersion was adjusted to give a BE of 932.62 eV for the Cu 2p3/2 line of metallic copper. The Kratos charge neutralizer system was used on all samples. Survey scan analyses were carried out on 0.05–0.1 g of sample, which was vacuum degassed for 24 h at 110 °C prior, with an analysis area of 300 × 700 microns and a pass energy of 160 eV. High-resolution analyses were carried out with an analysis area of 300 × 700 microns and a pass energy of 20 eV. Fitting was performed using CASA XPS (version 2.31), with the spectra being corrected to the main line of the carbon 1 s spectrum at 284.8 eV. 

Thermogravimetric (TGA) analysis was carried out with 10 mg samples on an SDT Q600 Simultaneous TGA/DSC from TA Instruments under air (50 mL min^−1^) at a scan rate of 10 °C min^−1^ from 80 °C to 600 °C. 

Viscosity was measured with the rolling ball viscometer LOVIS 2000 M/ME (Anton Paar Saint Laurent, QC, Canada). Samples (n > 2) of 0.006 g/mL were prepared and relative measurements with toluene at 30 °C at an angle of 45° were measured using the Solomon–Cuita equation (Equation (1)):

(1) $$\left[ƞ\right]=\frac{\left[2\left[\{\frac{t}{t_{0}}-\ln\left(\frac{t}{t_{0}}\right)-1\right\}\right]1}{2}/c$$

where *t* and *t*_0_ are the flow times of the solution and the solvent, respectively, and *c* is the concentration of the solution. 

The Mark–Houwink–Sakurada equation (Equation (2)) was used to determine viscosity average molecular weight (M) using reported *k* and *a* values [58]:

(2) $$\left[\eta \right]=k{M_{v}}^{\alpha }$$

## 3. Results and Discussion

### 3.1. Recovery of Polystyrene from Waste Food Containers

**Figure 1:**
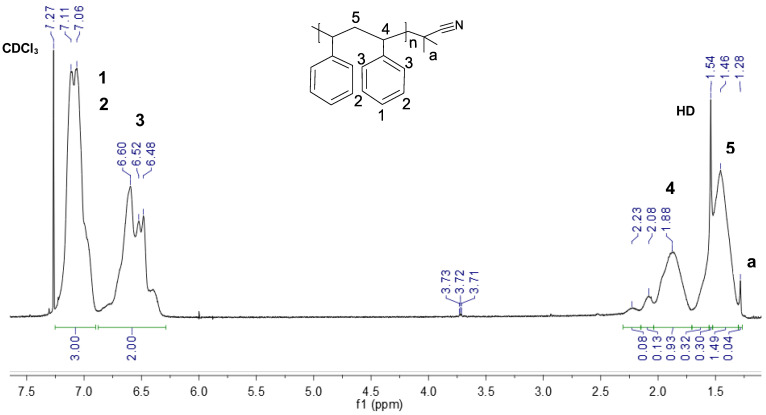
^1^H NMR spectrum of the recovered polystyrene from waste food containers.

Polystyrene was recovered using optimized dissolution/precipitation and reverse precipitation methods at ambient temperatures (Table S1 in the Supporting Information). In the former, the polystyrene solution in the methyl ethyl ketone solvent (MEK) was added to hexanes as described in the literature [9,11], but this resulted in a quick aggregation of the precipitated styrene with possible embedded impurities. This method also provided low yields compared to literature values [54] (Table S1). Reversing the method to add hexanes slowly dropwise to the PS/MEK solution provided fibrous PS (Figure S1). The MEK-to-hexanes ratio of 1:3.3 yielded the highest average percentage yield of 99.1% ± 0.006 for three replicates (Table S1) for the least amount of non-solvent used. This result indicates a 99.1% success in diverting waste from the landfills. The recovered PS was characterized by ^1^H NMR (Figure 1) and FTIR (Figure S2 in SI). 

Figure 1 shows that the recovered polystyrene is pure and shows all the characteristic peaks for the aromatic (3, 7.06–7.11 ppm and 1, 2 6.48–6.60 ppm), benzylic (4, 1.88 ppm), and aliphatic protons (5, 1.46 ppm). The spectrum also showed peaks for the sp^3^ protons with integration values (<1.0) for all samples at 1.28 ppm (a). This peak can be attributed to the end-group methyl protons emerging from the radical initiator azobis(isobutyronitrile) (AIBN) [59], which is commonly used for the polymerization of commercial polystyrene. The presence of this peak and the absence of low-intensity peaks of benzoate species between 7.4 ppm and 7.9 ppm also indicate that benzoyl peroxide—another commonly used initiator—was probably not used for this polymerization [60,61]. Notably, the triplet at 3.71 ppm indicates a peak for protons adjacent to alkoxy or alcohol groups, indicating that there may be some minor latent oxidative degradation in the recycled polystyrene that may trigger depolymerization during functionalization. However, this peak had an integration of <0.005.

**Figure 2:**
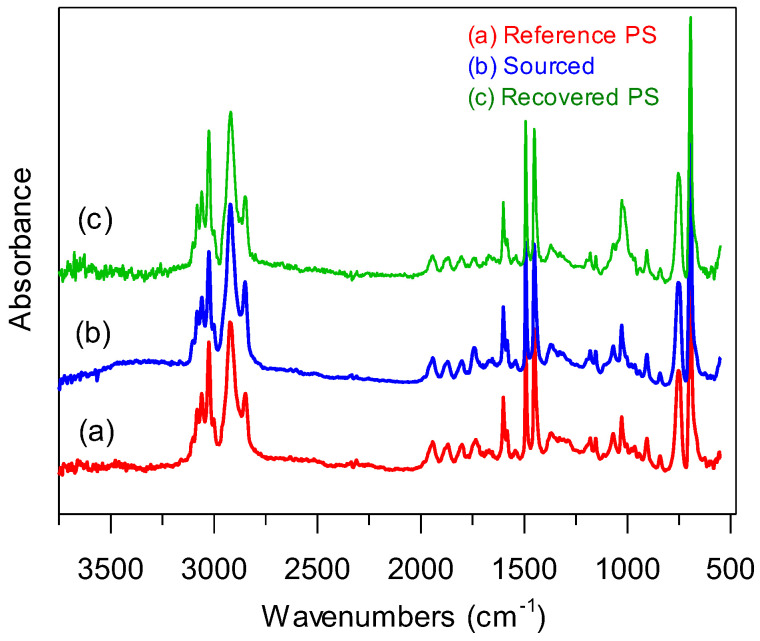
FTIR spectra for the (**a**) reference, (**b**) source, and (**c**) recovered PS samples.

Figure 2 shows the comparative FTIR spectra of the source PS packaging material, the recovered PS, and a reference PS. 

It is evident from Figure 2 that the polymer from the source sample (Figure 2b) is a PS homopolymer and was recovered successfully via chemical processing (Figure 2c), exhibiting all characteristic reference peaks for PS (Figure 2a). All spectra showed a medium-intensity peak for aromatic C-H stretching near 3060 cm^−1^, a weak overtone of the aromatic C-H bending between 1650 cm^−1^ and 2000 cm^−1^, a medium-intensity C=C stretch for the aromatic ring, 750 cm^−1^ and 690 cm^−1^ from C-H out-of-plane bending vibrations, and peaks near 1465 cm^−1^ for the benzylic and the methylene C-H bending of the polymer backbone. The source sample showed a broad peak near 3400 cm^−1^, which is attributed to residual absorbed water during the washing of the food containers. A strong C=O stretch near 1760 cm^−1^ and a broad peak near −3400 cm^−1^ for O-H stretching were absent in the recovered PS sample (Figure 2c), indicating that there was no obvious oxidative degradation and that the polymer was suitable for further modification. 

### 3.2. Polystyrene Functionalization

The recovered PS was reacted with formaldehyde and morpholine using the prepared Fe-doped TiO_2_ catalyst. The nitrogen adsorption (Figure S2a) showed a reversible type III isotherm indicating a macroporous structure with unrestricted multilayer formation and a specific surface area of 20.2 m^2^/g calculated by the BET equation. The pore widths ranged from 3 nm to 55 nm with a maximum at 11 nm (Figure S2b) characteristic of a mixed meso/macroporous material with a small pore volume (0.031 cm^3^/g). The anatase/rutile, meso/microporous structure has been known to enhance catalytic performance synergistically [62,63] compared to monolithic structures due to an improved mass transport. For the Mannich 3CR, aliphatic aldehydes and cyclic amines have shown efficient reactions with stable products produced at high yields [45,47,51]. Therefore, in this reaction with polystyrene via C-H activation catalyzed by meso/macroporous Fe-TiO_2_, formaldehyde and morpholine were used. Three ratios with recovered PS and an increasing aldehyde and amine content (1:5:5, 1:10:10, and 1:20:20) were synthesized, where the use of excess substrate for benzylic functionalization to obtain high yields with the Mannich reaction has been reported [50]. 

Sol–gel-derived Fe-SiO_2_ and Fe-TiO_2_ catalysts have been successfully used in A^3^, 3CR between various aldehydes, amines, and alkynes to form propargylamines via the C-H activation of the acidic hydrogen in alkynes (pKa 27) [45,46]. Transition-metal solid organic–inorganic catalysts used to accomplish the Mannich one-pot 3CR via C-H bond activation have also been reported for aldehydes and secondary amines with activated alkynes [64]. 

In the Mannich 3CR via C-H activation, regarding the bond dissociation energy (BDE) of the C-H bond to be activated, the electronic and steric environment around the C-H bond are important for transition-metal-mediated selective functionalization. The stabilization of the C-metal (C-M) intermediate by the surrounding electronic environment is also important for facilitating the insertion of the imine/iminium ion into the C-M bond [65]. The benzylic hydrogen that has the lowest C(sp^3^)-H BDE [52] conversely has a stronger C-metal bond energy [66], which is expected to further motivate this reaction. Also, the formation of the imine is independent of the C-H activation but has been generated in situ in a one-pot reaction using copper triflate as a Lewis acid catalyst for successful benzylic C-H activation [50]. Traditional Mannich reactions have required high temperatures maintained over long periods, generally 120 °C between 14–24 h, to achieve reasonable yields [50,51]. Therefore, the scope of this mechanism was evaluated for selective and energy-efficient functionalization of the recovered commodity atactic polystyrene in a solventless reaction facilitated by the sol–gel-derived organic–inorganic, solid, transition-metal catalyst under solventless, microwave conditions. 

**Scheme 1:**
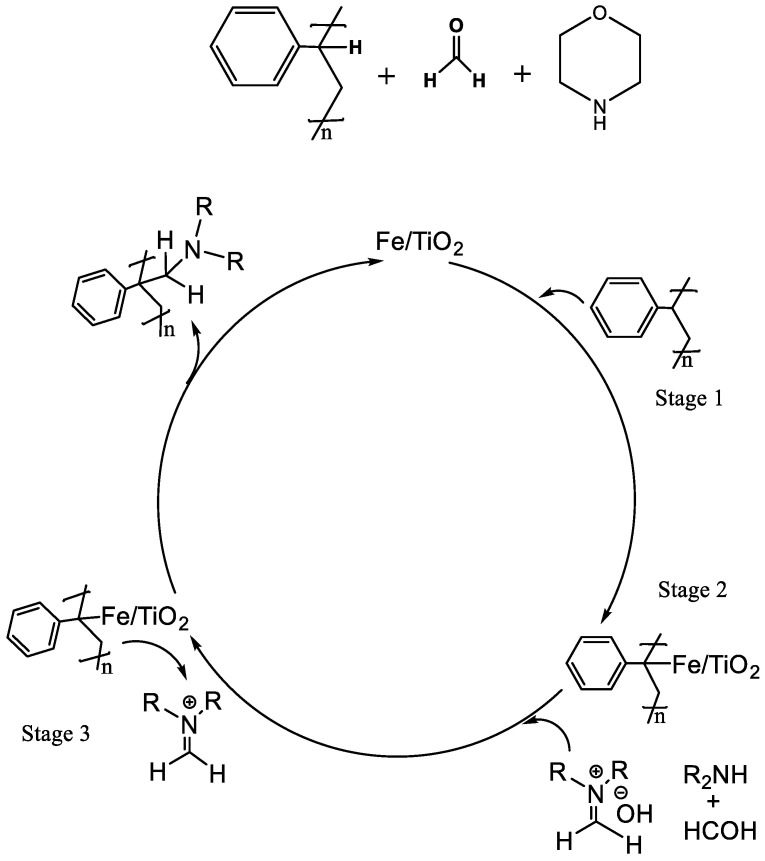
Proposed catalytic cycle of the three-component Mannich reaction catalyzed by sol–gel-derived Fe-TiO_2_ catalyst.

The tentative mechanism for the C-C coupling facilitated by the Fe-TiO_2_ involving the C-H activation of polystyrene at the benzylic position with the aldehyde and the secondary amine via a Mannich 3CR is shown in Scheme 1. 

The modified Mannich 3CR between the formaldehyde, morpholine, and benzylic C-H of the polystyrene proceeds through the C-H bond activation by the transition-metal catalyst, forming a R-C-Fe-TiO_2_ intermediate at the benzylic position (Stage 2, Scheme 1). This promotes the scission of the bond at this position for the subsequent C-C coupling step. The intermediate that is estimated to be stabilized by the π alkyne–metal complex in A^3^ reactions is proposed to be stabilized in this 3CR reaction via electron delocalization in the activated π aryl-Fe complex in the atactic polystyrene. This stabilized intermediate reacts with the iminium ion that is produced in situ (stage 2, Scheme 1) by the reaction of formaldehyde and morpholine (stage 3, Scheme 1) to form the functionalized polystyrene–amine product, regenerating the catalyst. 

A simultaneous and competing molecular depolymerization of the modified PS is also estimated to occur under the synergistic combination of the mixed anatase–rutile TiO_2_ catalyst that shows a higher dielectric heating efficiency under microwave irradiation, which potentially supports the 3CR while also promoting chain scission [67]. The use of transition metal oxides such as the Fe-based catalyst may also induce magnetic loss heating that generates additional heat [67,68]. Also, a mixed anatase–rutile catalyst (Degusa P-25) drives a two-fold increase in hydroxide radicals (•OH) in the presence of water [69] (e.g., from formaldehyde solution in water and alcohol) and under aerobic conditions. This is supported by non-thermal effects of MW irradiation [67,68,69] initiating polymer scission at existing weak linkages on the polymer chains as noted in Figure 1. 

## 4. Molecular and Structural Properties

### 4.1. Molecular Weight

The relative viscosity average molecular weight (M*_v_*) for commodity polystyrene (CPS), recovered PS, and the modified PS, MPS1–4, was obtained by solution viscometry and calculated using Equations (1) and (2), respectively. Table 2 shows the variation in the intrinsic viscosity and associated average molecular weights and yields of polystyrene before and after recovery and modification. 

The intrinsic viscosity (77.7 mL·g^−1^, Table 2) and the average molecular weight (1.82 × 10^5^ g·mol^−1^, Table 2) for the recovered polystyrene (PS) decreased marginally after recycling (77.1 mL·g^−1^, 1.80 × 10^5^ g·mol^−1^, Table 2), indicating that there was some fragmentation of polymer chains in agreement with other reported literature [54,70] despite ambient conditions. It is evident that, with the functionalization of the recovered material with small concentrations of formaldehyde and morpholine in MPS1, the number of atoms incorporated increases, registering an increase in intrinsic viscosity and average molecular weight (Table 2). The large decrease in intrinsic viscosity for MPS2 and MPS4 indicates that the modified polymers are significantly depolymerized under conditions of modification, forming functionalized oligomers [71]. Although this result indicates a successful functionalization and upcycling for MPS1, which has excess polystyrene substrate compared to the aldehyde and amine (Figure S4 in SI), for MPS2 and MPS4, where the F:M ratios are higher, it also incorporates increasing oxygen and nitrogen moieties, which can participate in hydrogen bonding, contributing to an increase in viscosity. Therefore, despite the significant fragmentation in MPS4, the recovery in intrinsic viscosity compared to MPS2 can be attributed to the higher hydrogen bonding content in the degraded polymer. MPS4 was obtained as a sticky and adhesive polymer (Figure S4 in SI), corroborating the enhanced hydrogen bonding. The yields of the polymers consistently decrease with an increasing modification but remain above an average of 85%, indicating the potential for optimizing conditions for scale-up. Also, the solid products are conveniently purified and separated from the catalyst, reducing tedious and solvent-intensive separation steps. 

### 4.2. Structural Characterization

**Figure 3:**
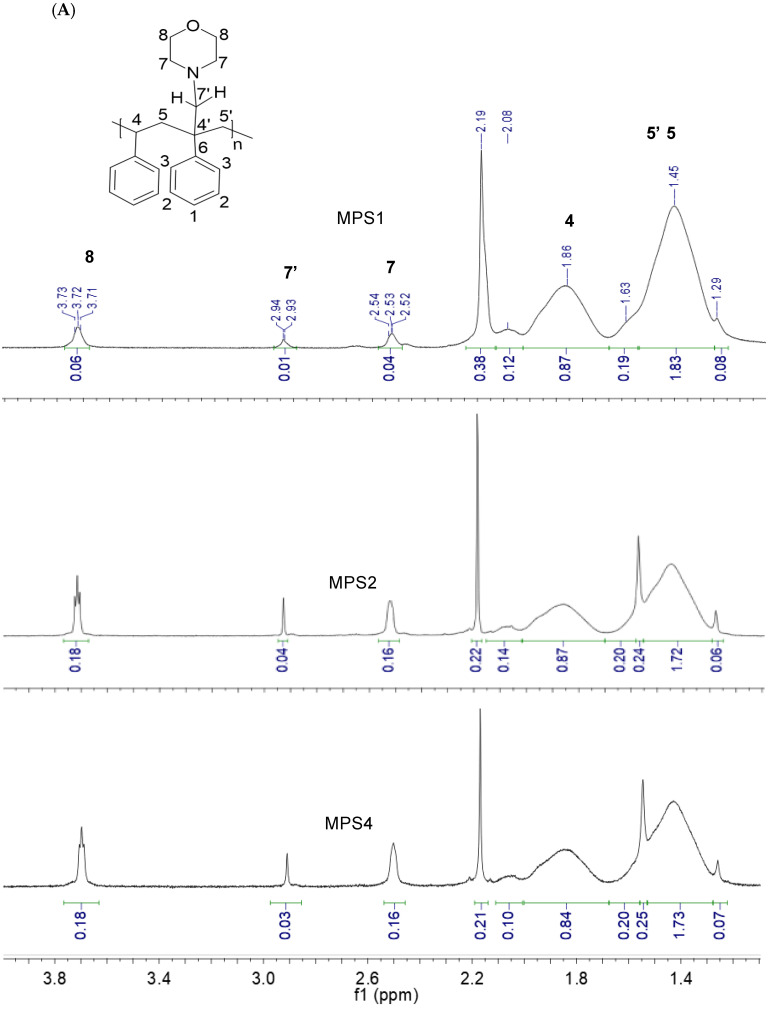

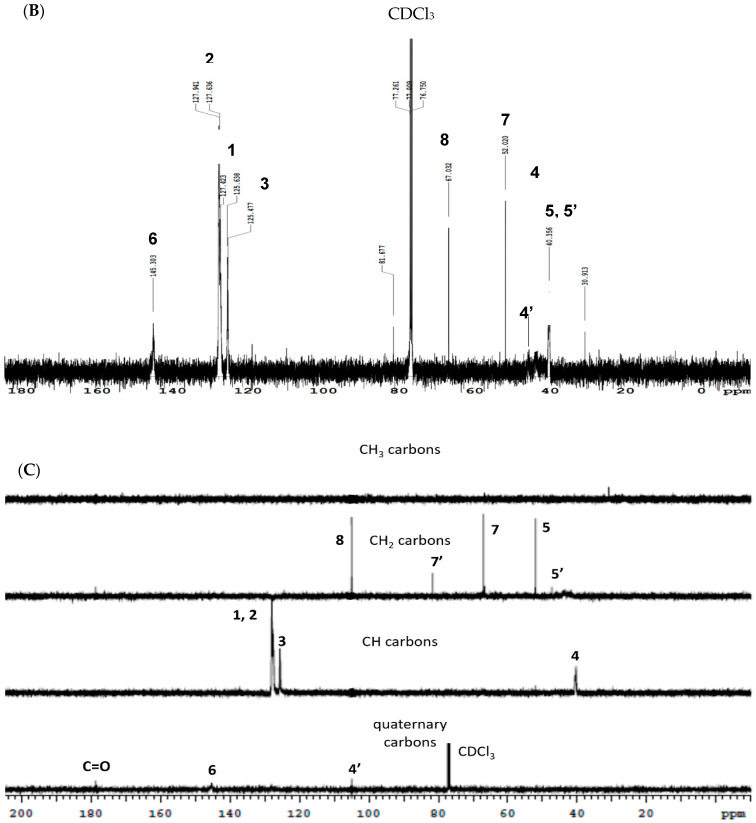
(**A**) ^1^H NMR spectra for the modified polystyrene ratios showing the functional region (**B**) ^13^C NMR spectrum of MPS4 and (**C**) DEPT ^13^C NMR spectrum of MPS4.

The ^1^H NMR spectra for the functionalized PS with varying ratios are shown in Figure 3A. Figure 3B,C show the ^13^C NMR and DEPT ^13^C NMR results for MPS4. Characteristic chemical shifts associated with PS (Figure 1) were present in all modified samples. The large singlet at 2.17 ppm was attributed to residual acetone trapped between the hydrogen-bonded chains after purification and drying. Chemical shifts for the four equivalent protons in the morpholine ring adjacent to nitrogen (N-C**H_2_**, 7 in Figure 3A) and oxygen (O-C**H_2_**, 8 in Figure 3A) are seen at 2.52 ppm and 3.71 ppm, respectively, indicating the successful incorporation of the morpholine ring in the modified polymers. The appearance of the new peak for the diastereotopic methylene protons (R-C-C**H_2′_**-N-(CH_2_)_2_-) formed further downfield at 2.93 ppm, 7′ in Figure 3A, confirms the formation of a new C-C bond at the benzylic carbon via C-H activation. This result indicates that the 3CR was successful and validates this approach toward functionalization. 

^1^H NMR was primarily used to ascertain a polymer structure, but it was also used to confirm if the enhanced viscosity (Table 2) was due to an increase in molecular weight or increased hydrogen bonding interactions. Interestingly, the NMR data revealed that there were no new peaks in the aromatic region of the modified polymers (Figure S5 in the SI) and that the J-values of the peaks in this region remained consistent from PS to MPS4 (0.05–0.12 ppm). This provided evidence that there was no substitution on the aromatic rings of PS and that the C-H activation selectively occurred at the benzylic position. Therefore, the number of protons of the aromatic ring (n = 5) was used as a reference for the integration of the other peaks for PS and the modified polymers (Figure 3A). The ratio of integration values enumerates 100 repeat units for MPS1. However, only 87 benzylic protons are integrated at 1.86 ppm, indicating an effective functionalization of 13% in MPS1. MPS2 and MPS4 showed a 12% and 15% modification at the benzylic position. Although this indicates increasing functionalization with an F:M ratio from MPS1 to MPS4, the increase is offset by polymer degradation. The consistently higher integration value at 3.71 ppm (CH_2_-O, 8 Figure 3A) compared to the value at 2.51 ppm (CH_2_-N, 7 Figure 3A) is attributed to the overlapping peaks (Figure 1) for CH_2_-O-R or other similar oxidized terminal derivatives that trigger degradation. This estimation is reasonable because, although modification is estimated to be between 12 and 15% for all ratios, a significant decrease of 46% is observed in viscosity from MPS1 to MPS2 (Table 2), which suggests that further C-C coupling in MPS2 and MPS4 is limited by polymer deterioration. Interestingly, no polymer deterioration is noticeable at 1.45 ppm (5, Figure 3A), which shows an almost consistent integration from MPS1 to MPS4. The integration of the chemical shifts for the ring protons (CH_2_-N) increases four-fold from MPS1 to MPS4, rising from 0.04 in MPS1 and plateauing at 0.16 in MPS2 and MPS4 (Figure 3A). This suggests that the reaction successfully ensues with the aldehyde and amine in stages 2 and 3 (Scheme 1) to integrate the entire ring structure on the polymer backbone but reaches a maximum extent of functionalization at MPS2. The integration for the chemical shift at 2.94 ppm for the new CH_2_ bond (7′, Figure 3A) arising from the C-C coupling reaction also increases three-fold from MPS1 to MPS4, evidencing a successful chemoselective C-H functionalization at the benzylic position constrained by polymer degradation. These results of functionalization described by ^1^H NMR are corroborated by the ^13^C NMR. The spectrum for MPS4, typical of the modified polymers but with larger oxidative degradation shown in ^1^H NMR, is shown in Figure 3B. The characteristic peaks of PS for the aromatic C1 and C2-C4 are seen at 140 ppm, 127 ppm, and 125 ppm, respectively. The benzylic carbon (4) appears at 45 ppm and the aliphatic carbon (5) is seen at 40 ppm [72]. The weak chemical shift for the quaternary functionalized benzylic carbon is evident at ~47 ppm (4′). The peaks for **C**-N (amine) and **C**-O (7, 7′ and 8, Figure 3B) indicating successful functionalization appear at 52 ppm, 55 ppm, and 67 ppm, respectively. Chemical shifts for carbons attached to oxidized species such as alcohols or esters indicating substantial oxidative degradation are seen at 82 ppm. The nitrile carbon from the initiator is observed at 110 ppm. The chemical shift at 120 ppm indicates that polymer degradation leading to alkene formation ensues in MPS4. Chemical shifts for methyl carbons (a) of the initiator or residual acetone and tertiary carbon group on AIBN appear between 20 and 30 ppm, respectively. DEPT ^13^C NMR was also used to confirm the formation of the quaternary functionalized benzylic carbon and the formation of the new CH_2_ bond due to the C-C coupling reaction. The chemical shifts for these carbons are shown in Figure 3C as 4′ and 7′ appearing at 82 ppm and 113 ppm, respectively. The presence of the carbonyl carbon at 180 ppm in MPS4 confirms oxidative polymer deterioration. Although the microwave irradiation in this reaction provided the activation energy to accelerate the formation of the aryl-Fe-metal complex and rapid imine formation, the increased temperature achieved from thermal and non-thermal MW irradiation effects proved to be contradictorily sufficient for C-H activation and for promoting polymer scission. Thermo-oxidative chain scission in polystyrene is initiated by a radical process that initially leads to the formation of alkyl macroradicals and reactive oxygen macroradicals (aliphatic C-O-O•) that subsequently form stable hydroperoxides (**H_2_**C-O-OH) as chain scission propagates [17,71]. These hydroxyl species further oxidize to carbonyl structures (C=O) such as carboxylic acids. This would explain the fragmentation of the polymers causing a decrease in viscosity in MPS2 and MPS4. 

**Figure 4:**
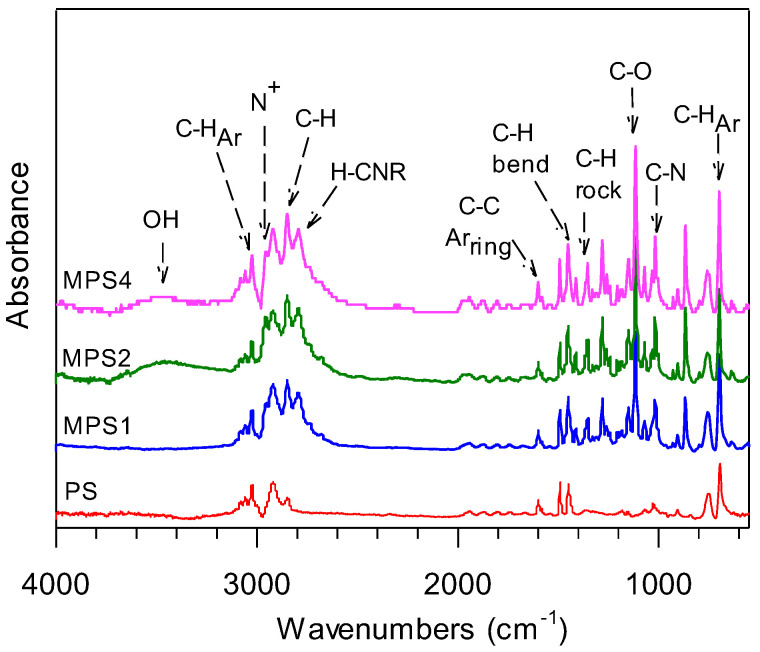
FTIR spectra for PS and the functionalized polystyrene polymers.

The formation of the selectively functionalized polystyrene product at the benzylic position by the Mannich 3CR reaction was also confirmed by ATR-FTIR (Figure 4). 

New medium-intensity peaks observed at 1020 cm^−1^ attributed to the C-N bending vibrations with the corresponding absence of the N-H bending vibration at 1580–1650 cm^−1^ in all MPS polymers allude to the successful integration of the functionalized tertiary amine on the PS backbone via an iminium intermediate following the Mannich mechanism. Interestingly, the FTIR spectra for all MPS samples show a peak with mildly incremental intensity from MPS1 to MPS4 for the N-H stretch associated with the formation of an amine salt (N^+^). The appearance of this peak confirms the formation of a tertiary amine due to successful C-C coupling which acts as a base in the presence of condensation emerging from the Mannich reaction. This result is significant because of the implications of the functionalized material in charge transfer or ion-exchange processes and applications. The appearance of the C-H stretches at 2789 cm^−1^, which are at a lower frequency than the C-H stretches of alkyl groups (2851 cm^−1^), indicate the formation of the new R-C-H alpha bond to nitrogen. This bond formation is also confirmed by the concurrent appearance and increase in intensity for peaks at 1353 cm^−1^ assigned to CH_2_ rocking vibrations in all functionalized polymers, also observed in amorphous regions in polyethylene [73]. An increase in the C-O peaks at 1112 cm^−1^ from MPS1 to MPS4 also supports the intramolecular transfer of the morpholine–iminium ion to the activated benzylic carbon of polystyrene with an increasing ratio. The O-H bending vibrations at 1410 cm^−1^ and the O-H stretching vibrations at 3469 cm^−1^ in the functionalized polymers indicate that the polymer chains are terminated due to oxidation/hydrolysis. These peaks are absent in PS and are weak in MPS1, but are most pronounced in MPS2 and MPS4, indicating an incremental rise in hydrogen bond density due to hydrolysis. These results support the results in Table 2 for the sustained intrinsic viscosity for MPS4. The presence of the hydroxyl region in FTIR also supports the higher integration of the chemical shift for CH_2_-O at 3.71 ppm attributed to the inserted ring protons but also from protons adjacent to latent and formed terminal hydroxyl groups. The absence of peaks near 1720 cm^−1^ for the carbonyl stretch in aldehydes, C=N bond vibrations near 1690 cm^−1^ for the imine intermediate, and a N-H stretch in morpholine confirm that the reactants have completely reacted.

**Figure 5:**
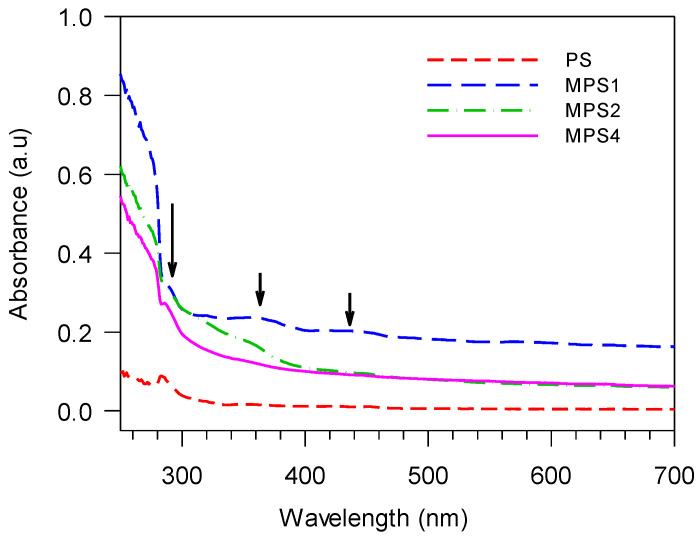
UV absorption spectra of the functionalized polystyrene polymers.

The comparative ultraviolet (UV) absorption spectra for polystyrene and the functionalized polymers are seen in Figure 5. 

The recovered PS shows a characteristic maximum absorbance at 285 nm, which is present in all functionalized polymers as well (large arrow in Figure 5). All functionalized polymers show a higher absorbance than PS but decrease in the order MPS1 > MPS2 > MPS4. MPS1 has the highest absorbance commensurate with the maximum functionalization of the polymer. Absorbance peaks at higher wavelengths—originating from interactions between electron-rich oxygen and nitrogen moieties in the functionalized polymer and the freely rotating phenyl structures of the PS chain [74]—are also observed clearly for MPS1 (small arrows in Figure 5). MPS2 and MPS4 show a broad shoulder at lower wavelengths, also emerging from similar but weaker interactions, which leads to a reduced overall absorbance. The UV spectral data confirm the ^1^H NMR and FTIR structural characterization that the most effective functionalization of PS via C-H activation in the multi-component Mannich reaction is achieved for MPS1. 

**Figure 6:**
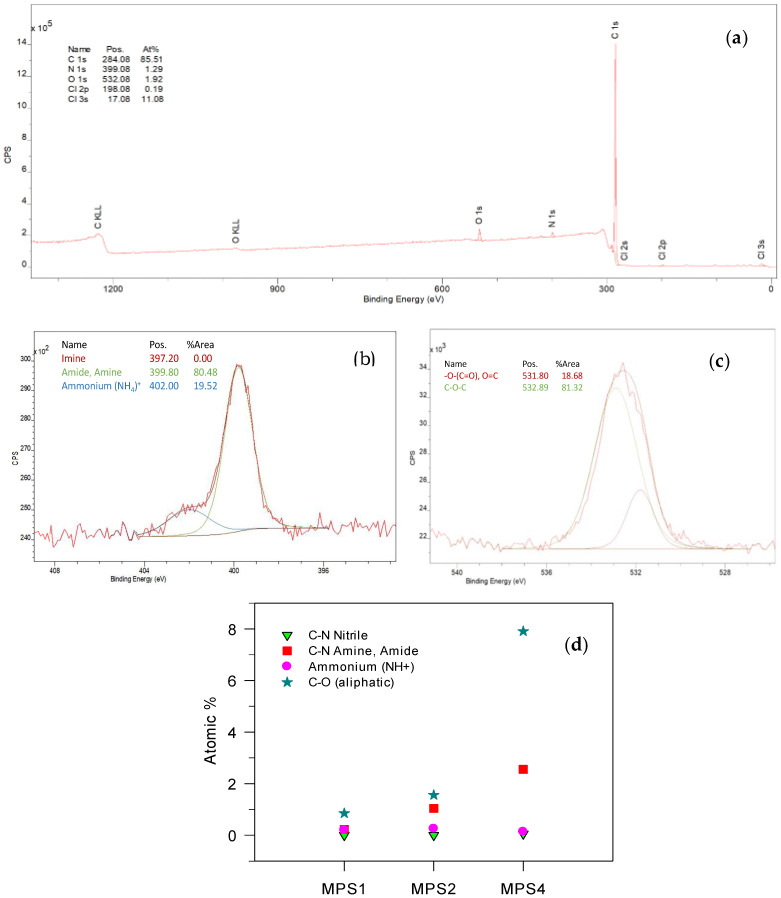
(**a**) XPS survey scan of MPS2, (**b**) the deconvoluted peaks for N 1s and (**c**) O 1 s spectra, and the (**d**) atomic percentage and speciation of carbon and nitrogen in the functionalized polymers.

The XPS survey scan for MPS2 shown in Figure 6a,b,c shows the deconvolution spectra of N 1s and O 1s, respectively, for MPS2, typical of the functionalized PS polymers. The atomic percentages of key elements (C 1 s, N 1 s, O 1 s) and their respective binding energies are shown in Figure 6d and listed in Table S3 in the Supporting Information. Chlorine seen in the survey scan (Figure 6a) denotes the likely presence of a residual amount from the catalyst in the sample after purification, or from the formation of the amine salt (Figure 4). Excess chlorine in MPS2 was mainly contamination originating from sample analyses conditions. The lower atomic percentage of nitrogen compared to oxygen is an indicator of the reduced functionalization compared to the relatively higher oxidative degradation in MPS2. The N 1s spectrum in Figure 6b is fitted with two peaks that clearly indicate the integration of the amine (N-C, 399.80 eV) through the C-C coupling reaction. The minor peak that appears at 400.2 eV in Figure 6b represents the formation of the N^+^ species in the functionalized polymer. The 80-percentage area ratio of the amine to ammonium in the sample indicates that most of the functionalized material is available as a tertiary amine that has the potential to be used in ion-exchange processes. Interestingly, the complete absence of the imine peak in the deconvoluted N 1 s spectrum confirms that the Mannich reaction goes to completion and is a valid mechanism for the selective post-polymerization functionalization of polystyrene. The O 1 s spectrum (Figure 6c) also exhibits an 80:20 ratio for the percentage area under the aliphatic O-C curve, commensurate with the 80-percentage area of N-C seen in Figure 6b. This confirms the successful integration of the morpholine ring in the functionalized polymers via the Mannich mechanism. Figure 6d shows the variation in the atomic percentage of carbon and nitrogen in the functionalized polymers. It is evident that the low but consistent amount (<0.04%) of N-C emerges from the nitrile group of the AIBN initiator, whereas the nitrogen content from the amine moiety N-C/N-C=O increases with an increasing F:M ratio (Figure 6d). MPS1 had the lowest atomic percent (0.43%), commensurate with the lowest F:M ratio, increasing to 1.29% in MPS2 and 2.69% in MPS4. This aligns reasonably with the four-fold increase (0.4 to 0.16) in the amine content by ^1^H NMR (2.52 ppm, peak 7, Figure 3A) and the increasing intensity of the C-N bending vibrations in FTIR at 1020 cm^−1^ in Figure 4. There is a simultaneous increase in the O-C content as well, but two things are significant here: (i) the oxygen content associated with O-C is always higher than the nitrogen content in all functionalized polymers and (ii) it is inordinately higher at the highest ratio (MPS4), indicating that oxidative degradation leading to the formation of peroxy/hydroperoxy species is promoted both at higher F:M ratios and at a lower polymer molecular weight in this reaction. It is also noteworthy that the functionalization of N and O content as seen by the atomic percentage is proportional to the F:M ratio only for MPS1 and then remains below double for MPS2 and below four times for MPS4, indicating that the functionalization itself was limited with an increasing ratio and supporting the conclusions drawn from NMR and IR. All functionalized polymers showed the formation of ammonium species that remained consistent in atomic percentage for all modified polymers, which demonstrates that the nitrogen cation formation was limited either by the water/chlorine content or was limited by a plateauing effect from the limited functionalization.

#### Thermal Transition Behavior

**Figure 7:**
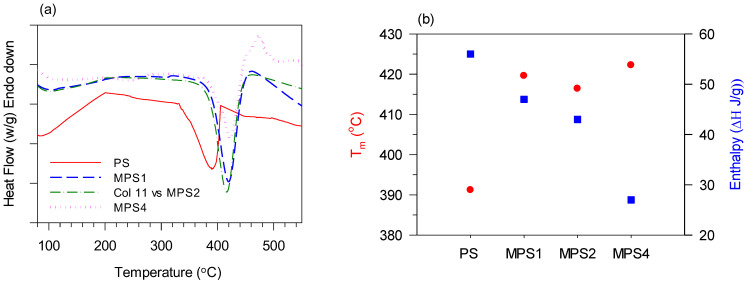
DSC thermograms obtained during the first heating cycle (10 °C/min) for PS and MPS with varying ratios, (**a**) peak temperatures of melting, and (**b**) enthalpy of melting.

The molecular structure and the molecular weight of polymers determine their thermal properties. Figure 7 shows the first DSC heating cycles of recovered PS and MPS with varying ratios.

All thermograms show a single melting transition indicating the presence of a single-phase microstructure in PS and the functionalized polymers, confirming that the additional functional components are integrated into the polymer system (Figure 7a). In comparison to the broader endotherm for PS, the sharper endotherms for the modified polymers indicate a greater interchain interaction, potentially due to the increased hydrogen bonding upon functionalization [75]. The shift in the melting temperatures from 391 °C (Figure 7b) to higher temperatures (417–423 °C) for the functionalized polymers (Figure 7b) is attributed to the increased molecular weight and secondary intermolecular bonding of the functionalized polymers. However, the consistent decrease in the enthalpy values from PS to MPS4 (Figure 7b) is attributed to the deterioration in chain size. MPS4 shows a curve characteristic of melting with decomposition [76]. The transition near 100 °C is possibly ascribed to the glass transition (Tg) temperature of polystyrene. 

### 4.3. Thermal Stability

**Figure 8:**
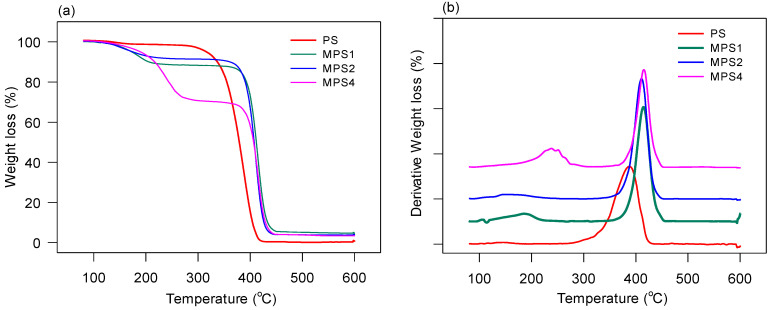
TGA curves for recovered PS and the MPS with varying ratios. (**a**) Percentage weight loss and (**b**) derivative percentage weight loss.

Figure 8a,b show percentage weight loss versus temperature and derivative thermogravimetric (DTG) plots, respectively, for the functionalized polymers.

The overall thermal decomposition temperature for these hydrocarbon-based polymers is around 380–420 °C, attributed to the C-C decomposition of the organic content. The functionalized polymers MPS1 to MPS4 show a higher overall decomposition temperature compared to the recovered polystyrene (Figure 8a), possibly due to the higher carbon content originating from the functional cyclic compounds. MPS1 showed the highest thermal stability of the functionalized polymers, which decreased in MPS2 and was the lowest in MPS4. The functionalized polymers also showed a residual 2–3.5% of inorganic or ash content. The DTG curves (Figure 8b) revealed a multi-step degradation typical of nitrogen and oxygen-containing polymers such as poly(ester urethanes) [75,77]. The weakest link is the C-N bond that ruptures around 200–230 °C, followed by the scission of the alkoxy oxygen bond (C-O) between 350 and 445 °C in the morpholine ring, which was overlapped by the decomposition of the C-C bonds above 380 °C. MPS4—which had the highest formaldehyde and morpholine content relative to polystyrene—showed the most pronounced decomposition peaks, which are shifted to the right due to the increased resistance to decomposition from the attached functional groups and the higher carbon content (R-**CH_2_**-NR_2_) introduced by C-C coupling. The onset temperature of decomposition for PS and the functionalized polymers measured at 95% mass loss (T_d(on5%)_) decreased with an increasing PS:F:M ratio. PS showed T_d(on5%)_ at 316 °C, which dropped between 173 and 175 °C for MPS1–MPS4. Such a decrease in thermal stability is attributed to the increasing content of the weaker C-N bond [75,77].

## 5. Conclusions

The first use of the Mannich three-component coupling reaction via the selective C-H activation of the benzylic position was reported for the post-polymerization and upcycling of waste commodity polystyrene recovered from food containers. Polystyrene was successfully diverted from the landfill and recovered with a 99.1% yield, with a viscosity average molecular weight of 1.80 × 10^5^ g·mol^−1^ and an estimated number average molecular weight M*_n_* of 45,970 following a reverse solution-precipitation method in a 1:3.3 methyl-ethyl-ketone: hexanes, solvent: non-solvent system. The Mannich reaction of recovered polystyrene (PS) with formaldehyde (F) and morpholine (M) was achieved using a sol–gel-derived Fe-TiO2 catalyst in a solvent-free, microwave-assisted synthesis. The microwave conditions contradictorily facilitated the selective C-C coupling selectively at the relatively acidic benzylic hydrogen, but also accelerated the oxidative deterioration of polystyrene with increasing functionalization. The integration of varying hydrogen bond densities in the modified polymers that compensated for the deterioration of molecular weight implies potential for tunable structure properties. The reaction mechanism was shown to be consistent and complete at higher functional ratios during the multicomponent reaction but was constrained due to the concurrent polymer degradation as confirmed by ^1^H NMR, ^13^C NMR, FTIR, XPS, and UV spectroscopies. The overall thermal stability of the functionalized polymers was higher than the recovered polystyrene. The integration of the quaternary ammonium cations indicated successful functionalization for all ratios, with the optimum functionalization observed for the lowest F:M ratio in MPS1, validating the use of this reaction for upcycling recovered polystyrene and for its potential use in ion-exchange membranes. 

## Figures and Tables

**Table 1:** Nomenclature and stoichiometric ratios for the polystyrene functionalization.

	Polystyrene (mol)	Formaldehyde (mol)	Morpholine (mol)	Fe-TiO_2_ Catalyst (g)
PS	1.7 × 10^−5^	0	0	0
MPS1	1.7 × 10^−5^	0.5 × 10^−2^	0.5 × 10^−2^	5.0 × 10^−2^
MPS2	1.7 × 10^−5^	1.0 × 10^−2^	1.0 × 10^−2^	5.0 × 10^−2^
MPS4	1.7 × 10^−5^	2.0 × 10^−2^	2.0 × 10^−2^	5.0 × 10^−2^

**Table 2:** Molecular weight and mass yields for polystyrene before and after modification.

	Intrinsic Viscosity (mL·g^−1^)	Average Molecular Weights (g·mol^−1^)	Yields (%)
CPS	77.7 ± 1.30	1.82 × 10^5^ ± 0.280	-
PS	77.1 ± 0.730	1.80 × 10^5^ ± 0.730	99.0
MPS1	78.3 ± 0.980	1.84 × 10^5^ ± 0.980	94.0
MPS2	48.9 ± 0.590	9.84 × 10^4^ ± 0.590	90.0
MPS4	59.0 ± 0.870	1.24 × 10^5^ ± 0.870	88.0

## Data Availability

Data is provided in the Supporting Information.

## Supplementary Materials

The following supporting information can be downloaded at: https://www.mdpi.com/article/10.3390/polym15143108/s1.
