# Supplementary material for: Circular Design and Functionalized Upcycling of Waste Commodity Polystyrene via C-H Activation Using Microwave-Assisted Multicomponent Synthesis
Source: Polymers (Basel). 2023 Jul 21;15(14):3108. doi: 10.3390/polym15143108 (PMC10384716; doi:10.3390/polym15143108)
Supplement: Supplementary file 1 [file polymers-15-03108-s001.zip › polymers-2514972-supplementary.pdf]

Supporting Information for:

Circular Design and Functionalized Upcycling of Waste Commodity Polystyrene via C-H Activation Using Microwave-Assisted Multicomponent Synthesis

Shegufta Shetranjiwalla [1,2,*], Claire Cislak [2] and Kevin M. Scotland [2]

1 School of Science and the Environment, Grenfell Campus, Memorial University of Newfoundland, Corner Brook, NL A2H 5G4, Canada
2 Chemistry Department, Trent University, Peterborough, ON K9K 0G2, Canada; clairecislak@trentu.ca (C.C.); kevinscotland@trentu.ca (K.M.S.)
* Correspondence: sshetranjiwalla@grenfell.mun.ca

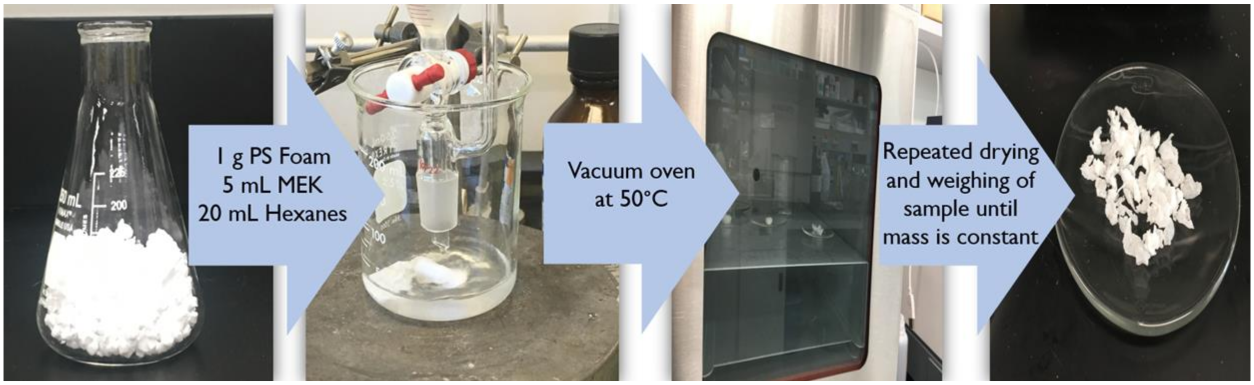

Figure S1. The optimized procedure for recovery of polystyrene from waste clamshell containers.

Table S1: Optimization of the dissolution/precipitation method (n=3)

Polystyrene (g)	MEK (mL)	Hexanes (mL)	MEK:Hexanes Ratio	Recovered Yield (%)
**Dissolution/Precipitation Method**				
1.00	5.00	20	1:4	81.4 ± 0.06
1.00	5.00	50	1:10	75.2 ± 0.43
1.00	5.00	100	1:20	82.3 ± 0.01
**Reverse Precipitation Method**				
1.00	5.00	50	1:10	77.9 ± 0.12
1.00	5.00	35	1:7	98.5 ± 0.003
1.00	5.00	25	1:5	99.5 ± 0.037
1.00	5.00	20	1:4	102
1.00	5.00	16.5	1:3.3	99.1 ± 0.006
1.00	5.00	15	1:3	98.5 ± 0.002

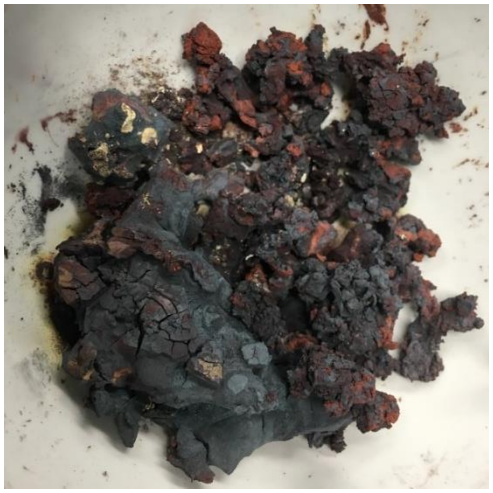

Image I1: Rutile and anatase mixed phases in the Fe/TiO_2 catalyst.

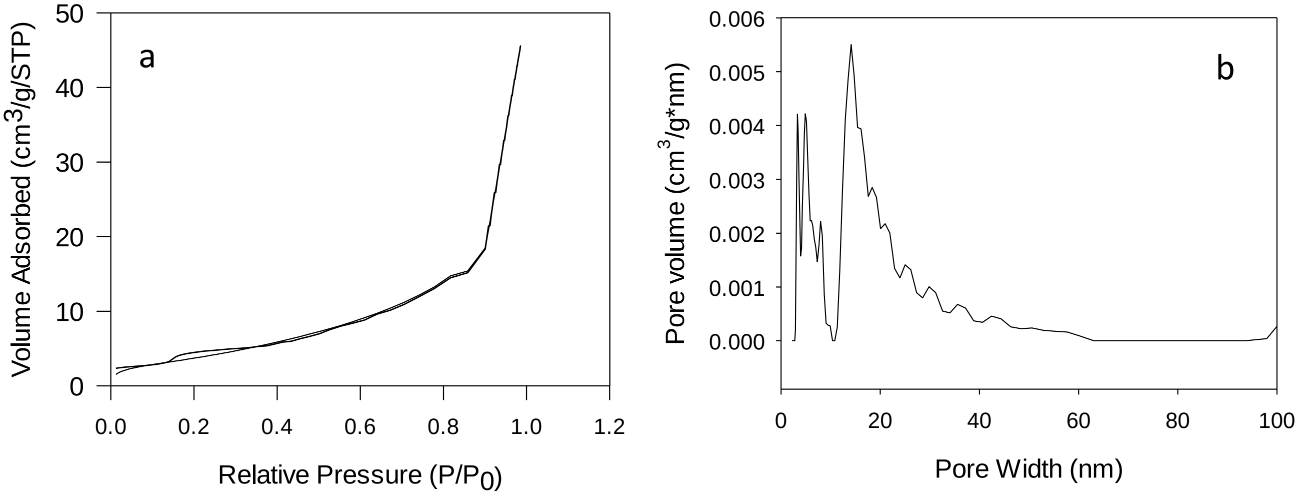

Figure S2. (a) N2 adsorption isotherm of Fe/TiO2 and (b) Fe/SiO2 along with corresponding pore size distribution derived from adsorption isotherm (inset

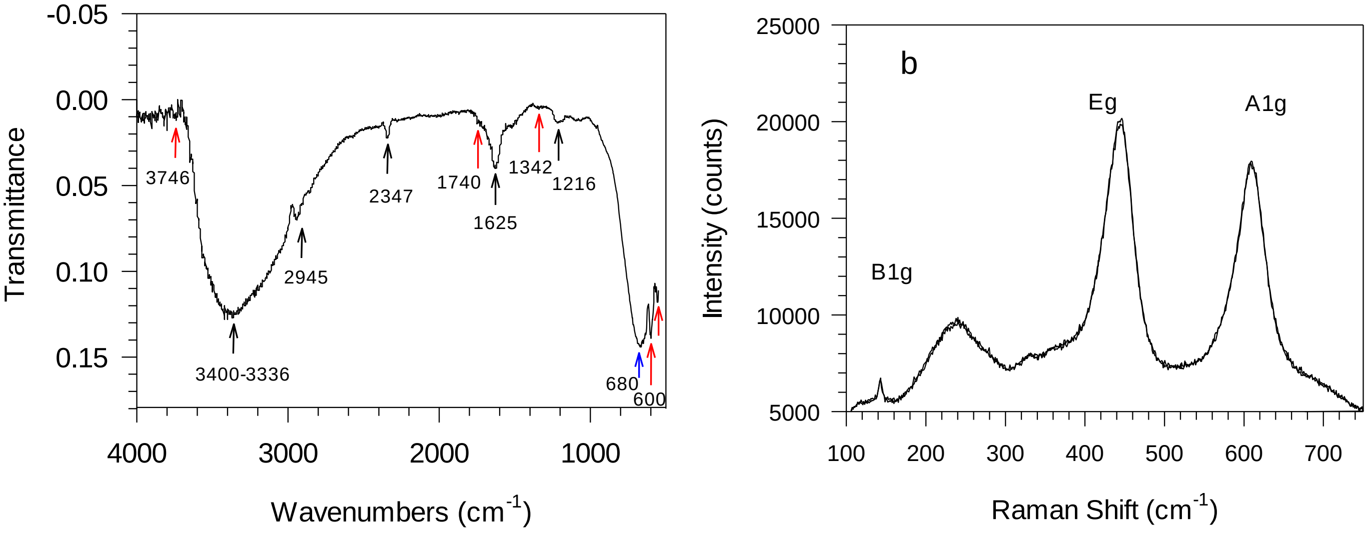

Figure S3. (a) FTIR spectrum of the Fe/TiO2 catalyst. The red arrows indicate the characteristic peaks for anatase phase, and the blue arrows indicates the characteristic peaks for the rutile phase. (b) Raman Spectrum for the red specs in the catalyst to show the rutile phase.

Table S2: FTIR peak assignments for the mixed anatase and rutile phases of Fe/TiO2 catalyst.

Peaks (cm^{-1})	Assignment	Phase		Reference
		Anatase	Rutile	
549	Ti-O	+	-	[1]
600	Ti-O	+	-	[1]
680	Ti-O-Ti	-	+	[1]
1216	C-O	+	+	
1625	O-H bending of molecularly adsorbed H2O	+	+	[2]
1740	Titanium carboxylates	+	-	[1,3]
2945	C-H	+	+	
3366	Ti-OH stretching	+	+	[1,3]
3400	O-H stretching			[1-3]
3746	Free O-H	+	-	[2,3]

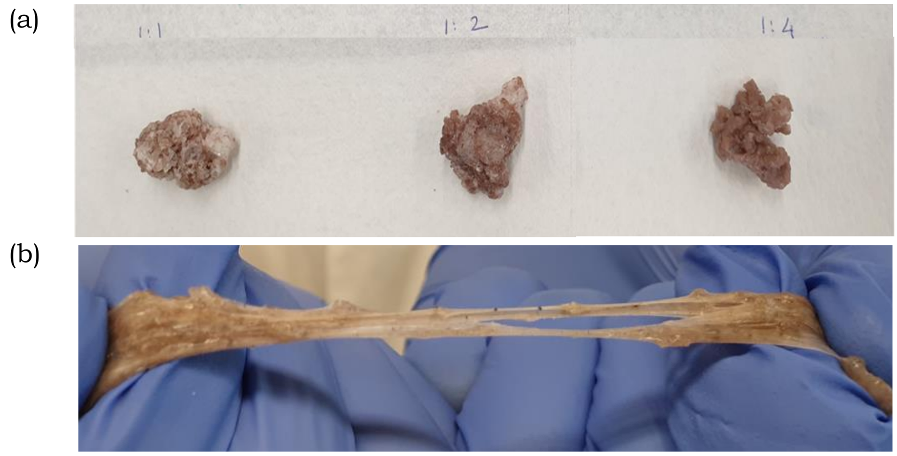

Figure S4. (a) Integration of F:M ratios with recovered PS and (b) adhesive nature of MPS4

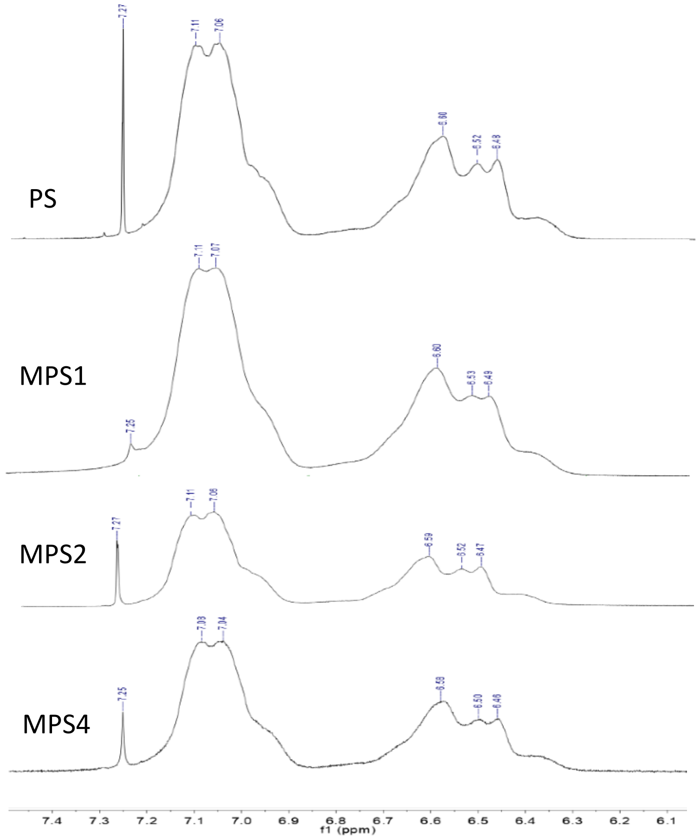

Figure S5: ^{1}H NMR chemical shifts and J-values for the aromatic region of PS and the various ratios of the functionalized polymers

Table S3: XPS data for the C1s, N1s and O1s of the functionalized polystyrene ratios

	MPS1		MPS2		MPS4	
	Binding Energy (e.V)	Atomic (%)	Binding Energy (e.V)	Atomic (%)	Binding Energy (e.V)	Atomic (%)
**C1s**						
C-C, C-H	284.91	96.99	284.93	93.60	284.94	82.22
C=N/C-N/C-O	286.63	0.80	286.62	1.79	286.64	4.41
C=O	287.73	0.83	287.30	1.37	287.74	2.35
C1s total:		*98.62*		*96.76*		*88.98*
**N1s**						
N≡C/C=N	397.30	0.00069	397.20	0.00	397.28	0.0547
N-C, N-C=O	399.89	0.23	399.80	1.04	399.88	2.56
$-NH_3^+$	402.10	0.20	402.00	0.252	402.08	0.138
N1s total:		*0.43*		*1.29*		*2.75*
**O1s**						
O-(C=O), O=C aliphatic	532.40	0.087	531.80	0.359	531.10	0.355
O-C (aliphatic)	532.85	0.085	532.89	1.56	532.45	7.92
O1s total:		*0.17*		*1.92*		*8.28*

REFERENCES

1. El-Sherbiny, S.; Morsy, F.; Samir, M.; Fouad, O.A. Synthesis, characterization and application of TiO2 nanopowders as special paper coating pigment. *Applied Nanoscience* **2014**, *4*, 305-313.
2. Suda, Y.; Morimoto, T. Molecularly adsorbed water on the bare surface of titania (rutile). *Langmuir* **1987**, *3*, 786-788.
3. Kotadia, D.A.; Soni, S.S. Stable mesoporous Fe/TiO2 nanoparticles: A recoverable catalyst for solvent-free synthesis of propargylamine via CH activation. *Applied Catalysis A: General* **2014**, *488*, 231-238.
